# Delivery of supported self‐management in remote asthma reviews: A systematic rapid realist review

**DOI:** 10.1111/hex.13441

**Published:** 2022-04-11

**Authors:** Emma Kinley, Imogen Skene, Elizabeth Steed, Hilary Pinnock, Kirstie McClatchey

**Affiliations:** ^1^ Asthma UK Centre for Applied Research, Usher Institute The University of Edinburgh Edinburgh Scotland; ^2^ Asthma UK Centre for Applied Research, Centre for Primary Care, Wolfson Institute of Population Health Queen Mary University London London England

**Keywords:** asthma, primary care, PRISMS taxonomy, remote consultation, supported self‐management, telephone consultations, video consultations

## Abstract

**Background:**

The COVID‐19 pandemic forced health care systems globally to adapt quickly to remote modes of health care delivery, including for routine asthma reviews. A core component of asthma care is supporting self‐management, a guideline‐recommended intervention that reduces the risk of acute attacks, and improves asthma control and quality of life.

**Objective:**

We aimed to explore context and mechanisms for the outcomes of clinical effectiveness, acceptability and safety of supported self‐management delivery within remote asthma consultations.

**Design:**

The review followed standard methodology for rapid realist reviews. An External Reference Group (ERG) provided expert advice and guidance throughout the study. We systematically searched four electronic databases and, with ERG advice, selected 18 papers that explored self‐management delivery during routine asthma reviews.

**Setting, Participants and Intervention:**

Health care professional delivery of supported self‐management for asthma patients during remote (specifically including telephone and video) consultations.

**Main Outcome Measures:**

Data were extracted using Context‐Mechanism‐Outcome (C‐M‐O) configurations and synthesised into overarching themes using the PRISMS taxonomy of supported self‐management as a framework to structure the findings.

**Results:**

The review findings identified how support for self‐management delivered remotely was acceptable (often more acceptable than in‐person consultations), and was a safe and effective alternative to face‐to‐face reviews. In addition, remote delivery of supported self‐management was associated with; increased patient convenience, improved access to and attendance at remote reviews, and offered continuity of care.

**Discussion:**

Remote delivery of supported self‐management for asthma was generally found to be clinically effective, acceptable, and safe with the added advantage of increasing accessibility. Remote reviews could provide the core content of an asthma review, including remote completion of asthma action plans.

**Conclusion:**

Our findings support the option of remote delivery of routine asthma care for those who have this preference, and offer healthcare professionals guidance on embedding supported self‐management into remote asthma reviews.

**Patient and Public Contribution:**

Patient and public contribution was provided by a representative of the Asthma UK Centre for Applied Research (AUKCAR) patient and public involvement (PPI) group. The PPI representative reviewed the findings, and feedback and comments were considered. This lead to further interpretations of the data which were included in the final manuscript.

## 1 INTRODUCTION

There are 339 million people living with asthma worldwide.^1^ Asthma is a variable condition and evidence‐based guidelines (e.g., GINA^2^; BTS/SIGN^3^) highlight the importance of supporting people to recognize when their condition is deteriorating and to know how to adjust their treatment and/or seek medical advice in a timely and effective manner. Supported self‐management is an approach that facilitates patients with long‐term conditions (LTCs; such as asthma) to have the knowledge, skills, and confidence to manage the physical, emotional and social impact of their condition(s).^4^ The ‘overwhelming’ conclusion of evidence syntheses is that supported self‐management for asthma improves asthma control, reduces exacerbations and hospital admissions, and improves patients' quality of life.^5^, ^6^ Despite this robust evidence, implementation in routine clinical care is challenging.^7^

Remote consulting, already promoted as a partial solution to growing challenges of healthcare delivery, was rapidly expanded in response to the worldwide COVID‐19 pandemic.^8^ Within the United Kingdom general primary care, there was a dramatic shift away from face‐to‐face consultations to telephone, video and on‐line consultations.^9^ In the months following UK COVID‐19 lockdown in early 2020, only 11% of primary care general practice appointments were conducted face‐to‐face, suggesting that nearly 90% of patient‐provider interactions took place via remote means.^10^ Remote consultations can potentially provide both benefits and challenges for patients and health professionals. Suggested advantages of remote consulting include improving access to care for LTCs,^11^ maximizing the potential for supporting self‐management,^12^ overall acceptability, safety and effectiveness^13^, ^14^ and improvements in asthma control.^15^, ^16^ However, critics have raised concerns about the use of remote delivery of routine primary care due to variable evidence of suitability and the associated technical, clinical and organisational policy challenges.^17^

### 1.1 Rationale for review

Published research regarding the delivery of supported self‐management during remote asthma consultations is sparse and the speed of technological advance means it needs frequent updating. Given the changing clinical context and national/international recommendations for implementing supported self‐management,^2^, ^3^ providing guidance on this new approach to delivery is timely. Informing a UK‐wide cluster randomized controlled trial, evaluating the implementation of supported self‐management (IMPlementing IMProved Asthma self‐management as RouTine [IMP^2^ART]), this study uses a rapid realist review approach^18^, ^19^, ^20^, ^21^ to explore the clinically effective, safe and acceptable delivery of supported self‐management of asthma via remote routine reviews. Conducting a rapid realist review will enable understanding not only about whether an intervention/approach works but how and in what clinical, demographic or organisational context.

### 1.2 Study objectives

Using realist methodology, we aimed to:

1. Identify and synthesize studies that evaluated and/or explored remote asthma consultations and the delivery of supported self‐management.
2. Explore the context and mechanisms that have contributed to clinically effective, safe and acceptable delivery of supported self‐management during remote asthma consultations.
3. Produce recommendations for best practices in the delivery of supported self‐management during remote consultations for people with asthma.

## 2 METHODS

### 2.1 Study design

Following Realist Review methodology^18^, ^19^, ^20^, ^21^ to identify Context–Mechanism–Outcome (C–M–O) configurations within existing research, our review explored how supported self‐management is delivered during routine remote asthma reviews. The study is reported in line with the RAMESES Publication Standards for Realist Synthesis and Realist Reviews.^22^ We registered our protocol on the PROSPERO database (Registration No.: CRD42020207543).

### 2.2 Realist methodology

Devised by Pawson and Tilly,^18^, ^20^, ^21^ realist methodology is a theory‐driven review process that focuses on understanding the interplay of an intervention's Context (C), Mechanisms (M) and Outcomes (O) and whether the intervention works (or not). Conducting realist research aims to answer the question ‘what works, for whom, in what contexts, to what extent and most importantly how and why’.^18^ Realist methods are increasingly used within healthcare research due to their ability to support the understanding of complex interventions. Within realist methodologies, a programme theory is a specific hypothesis about how an intervention causes the intended or observed outcomes and should be the central aspect of any realist evaluation or synthesis.^23^ Several varied theories are identified initially using a broad scope of existing literature to refine the purpose of the review and identify review questions. Programme theory formulation is subsequently an iterative process that progresses as the evidence is identified, assessed and synthesized, until an evidence‐based saturation and conclusion have been reached.^20^ Pawson differentiates between realist evaluations—an approach used when conducting primary research, and realist synthesis—an approach used to synthesize secondary data.^20^

### 2.3 Rapid realist review

**Figure 1:**
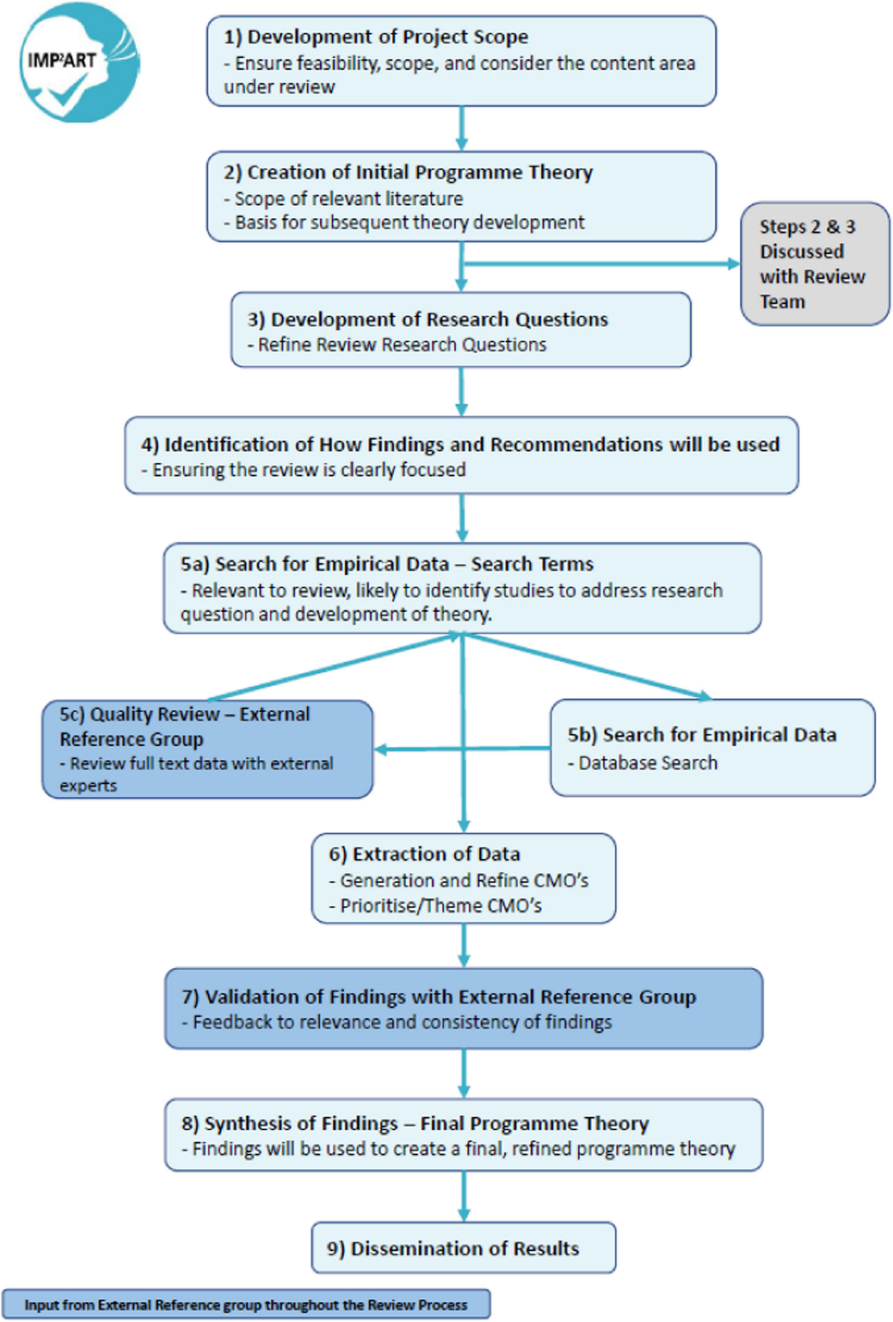
The rapid realist review approach (adapted from Saul et al.)^19^

For this review, we used the rapid realist review approach described by Saul et al.^19^ (displayed in Figure 1) to apply a realist synthesis approach in a timely manner where there is an emerging evidence base for the subject area under review, while still preserving the core elements of realist methodology. An important feature of a rapid realist review is to engage an External Reference Group of experts, who provide informed direction to the data identification and theory development throughout the entirety of the review, and ensure the review is grounded in the local context. We convened a multidisciplinary External Reference Group, including researchers, clinicians (nurses and GPs) and primary care respiratory experts. Members met twice during the review process, initially to provide feedback on the project scope and the full‐text articles proposed for inclusion. The group met again to review findings from the data extraction and advise on data synthesis.

### 2.4 Scoping the literature

The review took place between August 2020 and March 2021. One reviewer (E. K.) initially scoped relevant literature exploring the delivery of routine asthma reviews via remote consultations. From this initial broad search, the research group created a preliminary programme theory. This process defined the scale of the research and ensured that the review focussed appropriately on the research questions. Although not an essential element of a rapid realist review, the creation of programme theory is a recognized step in realist approaches.^18^, ^19^, ^20^, ^21^ We, therefore, decided to adopt this approach within this review: The initial programme theory formed the basis of the iterative data collection, data extraction, data synthesis and subsequent theory development stages, and the concluding programme theory allowed a final statement of the evidence to be produced.

### 2.5 Search process

The following databases were searched in October 2020 by E. K.: MEDLINE, Embase, PsychINFO and the Cochrane Library. Key search terms that were likely to identify studies relevant to the research questions and to address the purpose of the review were used (File S1). We searched for qualitative, quantitative, mixed‐method studies and grey literature published after 2000, to reflect contemporary remote consultation technologies, and the introduction in the UK of the Quality Outcomes Framework in 2004 (which incentivized regular reviews for LTCs including asthma).^24^ Using the PICOS Framework,^25^ eligibility criteria were developed (Table 1), and studies that did not meet the inclusion criteria or were not published in the English language were excluded. Consistent with a realist synthesis approach, it was still possible for data beyond this framework to be included in the review if the article contributed to the development of the review's programme theory. Documents were assessed collectively by the study team to determine whether the evidence provided was ‘good enough and relevant enough’,^21^ to inform the creation of appropriate C–M–O configurations within the data. In line with the iterative approaches of realist methodologies, we used snowballing techniques (such as searching companion papers and citation tracking) for all included articles to ensure that important texts were not overlooked. We also searched for additional relevant grey literature (e.g., policy documents, opinion pieces) from a variety of sources (including any suggested by the External Reference Group). The search process was iterative, overlapped with data extraction and analysis, and was directed towards the evidence gaps and finding explanatory information.

**Table 1:** Inclusion and exclusion criteria for the rapid realist review systematic database search, following the PICOS Framework^25^

	Inclusion	Exclusion
**P** opulation	Adults or children with a diagnosis of asthma	Participants with other long‐term health conditions (unless the study presented data for people with asthma separately)
	Healthcare professionals who regularly deliver asthma care	
		Patients receiving care under severe asthma clinics (because they have specialist needs which may be different to the majority of primary care patients)
**I** ntervention	Remote consultations (e.g., telephone/video consultations)	Studies that use automated telehealth interventions (e.g., mobile apps or email consultations) and do not include personalized contact with a healthcare professional in real time
	Includes delivery of supported self‐management	
		Interventions targeted at people under a severe asthma clinic
		No remote consultation delivery
**C** omparison	Trials that compare remote asthma care consultations versus standard face‐to‐face (in‐person) reviews	Trials that do not compare remote asthma care consultations versus standard face‐to‐face (in‐person) reviews
	Before and after studies, assessing the implementation of remote reviews	
**O** utcomes	Delivery of a standard primary care asthma review	Studies that do not present any form of self‐management support
**S** tudy design	Quantitative, qualitative and mixed‐method studies	Studies that do not meet the study design inclusion criteria

### 2.6 Selection and appraisal of documents

Titles/abstracts and potentially eligible full texts were independently screened by two reviewers (E. K. and  I. S.), and disagreements were resolved by discussion.

It was during this stage, that the first External Reference Group Meeting took place (November 2020) to review the list of full‐text articles and provide feedback on the importance of included papers and suggest any other publications or research that might contribute data to the review. During this stage, there was also an emphasis on grey literature or ‘difficult to find’ documents which may have not otherwise be identified. From the feedback provided by the group, any gaps in the literature were addressed by iteratively modifying the search terms/inclusion and exclusion criteria to capture any further relevant documents.

### 2.7 Data extraction

To begin the data extraction phase, a template was devised focusing on C–M–O configurations that explored components of support for self‐management, as defined by the Practical Systematic Review of Self‐Management Support (PRISMS).^26^ The PRISMS taxonomy was used as a framework during the data extraction phase by categorizing C–M–Os into one of the 14 components of self‐management support, to streamline the subsequent data synthesis processes. Examples of these components include ‘A1. Information about condition and/or its management’ and ‘A2. Information about available resources’. PRISMS components are further explained within File S2. The data extraction template also allowed for recording as to whether each C–M–O related to the ‘acceptability, safety and clinical effectiveness’ of supported self‐management delivery during routine remote asthma consultations, in line with the project's aims and objectives.

C–M–O configurations were then extracted from all full‐text articles. Quantitative, qualitative or contextual data could be extracted from any part of selected papers. We continuously considered the relevance and rigour of each included C–M–O, and regularly discussed within the core research team (E. K., K. M., H. P. and L. S.) how individual extracts should be used to ensure appropriate inferences were made. Data extraction was completed by E. K. (25% was independently extracted by I. S. to ensure consistency of approach, reliability and validity. The independent extraction of data by the two authors (E. K. and I. S.) resulted in the same C–M–O configurations being extracted by both authors, that is, each author identified the same outcome related to remote delivery of SSM in all papers and also connected the same context and mechanism to each outcome for all papers. How to optimally present the data (i.e., which data extract was used) and the interpretation of each C–M–O was discussed in detail until a consensus was reached.

### 2.8 Analysis and synthesis processes

We used the PRISMS taxonomy^26^ to structure our synthesis. The PRISMS meta‐review highlighted the importance of supported self‐management as a key component of high‐quality care for people living with LTCs, concluding that healthcare providers should promote a culture of actively supporting self‐management as a routine, expected and monitored aspect of care.^26^ Self‐management is a broad concept applicable to different demographics of people living with a wide range of LTCs, and thus the support that can be provided is diverse. The use of the PRISMS taxonomy ensured we captured this breadth in a structured way.

To synthesise the findings, all extracted C–M–Os were mapped against the PRISMS taxonomy components. We considered C–M–Os for each component of self‐management and identified key themes within and across each component. Further, we considered whether there was variance in the frequency of delivery of each component. Following this, we considered the association of C–M–Os to the outcomes of acceptability, clinical effectiveness and safety. Key themes were created from all C–M–O and taxonomy components until data saturation was reached. As External Reference Group members included clinicians currently delivering supported asthma self‐management, their feedback ensured that the final findings and themes addressed any gaps in practice that the analysis had not represented.

## 3 RESULTS

### 3.1 Selection of included studies

**Figure 2:**
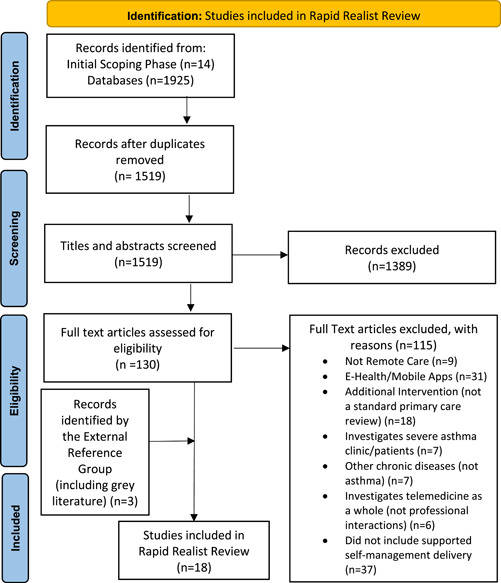
Search strategy and results^42^

A total of 1519 articles were identified in the search, of which 15^12^, ^17^, ^27^, ^28^, ^29^, ^30^, ^31^, ^32^, ^33^, ^34^, ^35^, ^36^, ^37^, ^38^, ^39^ met the inclusion criteria and were included in this rapid realist review. The External Reference Group identified an additional three papers.^14^, ^40^, ^41^ Although these papers did not meet the PICOS inclusion criteria (two papers had only recently been published so was therefore missed within the initial search, and the other paper focused on telemonitoring rather than in‐person self‐management delivery), they were still included due to their relevance to the review's aims. The PRISMA flow diagram^42^ illustrates the search strategy and results (Figure 2).

### 3.2 Study characteristics

All 18 included studies were published between 2003 and 2020 and were undertaken in the United Kingdom (*n* = 10), the United States of America and Canada (*n* = 7) and Italy (*n* = 1). Eight of the included papers were systematic reviews (*n* = 5) or meta‐reviews (*n* = 3), including data from a total of (*n* = 366) unique primary studies represented within these systematic reviews. Eleven papers had the primary aim of exploring the use of remote consultations in routine asthma reviews. Of the three papers provided by the External Reference Group, one was a feasibility study and two were systematic reviews. Detailed study characteristics can be found in File S2.

### 3.3 Main findings

The data extraction process was completed for the 18 included articles (full C–M–O configurations can be found in File S3). The PRISMS supported self‐management components most commonly informed by C–M–O configurations were:

1. A4: Regular clinical reviews.
2. A1: Information about the condition and/or its management.
3. A5: Monitoring of condition with feedback.
4. A3: Provision of/agreement on specific clinical action plans and or rescue medication.
5. A8: Provision of easy access to advice or support when needed.

These components, in addition to other self‐management strategies, have been explored through the data synthesis stage. Six key themes were identified which are described below, with an overarching C–M–O to outline the key conclusions of each theme (Table 2). Each theme presents findings from both an asthma patient and healthcare professional perspective, in addition to differences between the use of telephone and video consultations. Data saturation was reached for all themes.

**Table 2:** Findings: Six Key Themes

Findings: Key themes	Context–Mechanism–Outcomes (C–M–O) configurations		
	Background information e.g., setting and demographics to outline possible Contextual factors	Key workings that contributed to the design and functioning of a pathway to identify Mechanisms and resources	Information and evidence suggestive of the successes or failures of different aspects of an intervention (Outcomes)
1. Increased regular patient attendance and increased monitoring of patient	People with asthma scheduled for a routine review…	…who were provided with a review via telehealth technology (telephone/video consultation)…	…were more likely to attend their routine asthma review, and attend subsequent routine remote reviews.^14^, ^17^, ^27^, ^29^, ^30^, ^31^, ^32^, ^33^, ^34^, ^36^, ^39^
	Healthcare professionals (HCPs) conducting routine asthma reviews…	…via telephone/video consultation, with patients regularly attending routine remote reviews…	…have more opportunities to monitor a patient and provide regular support for self‐management.^27^, ^28^, ^30^, ^34^, ^38^, ^39^
	People with asthma whose routine review is conducted remotely…	…via video consultation…	… can have their condition and management successfully monitored, and the use of video can lead to increased patient attendance to regular routine reviews.^27^, ^30^
	People with asthma, whose routine review is conducted remotely…	…via telephone consultation…	… can have their condition and management successfully monitored, and the use of the telephone can lead to increased patient attendance to regular routine reviews.^34^, ^39^
2. Opportunities to provide individualized information about asthma and asthma management	People with asthma who are scheduled for a routine asthma review…	…via telephone/video call, in discussion with their health professional…	…can be provided with individualized information about their asthma, including education and principles of managing their condition.^27^, ^31^, ^32^, ^33^, ^35^, ^37^, ^38^, ^39^, ^40^, ^41^
	HCP conducting a routine asthma review…	…when the review is conducted via remote technologies…	…are able to deliver individualized information about a patient's condition safely and effectively.^27^, ^31^, ^32^, ^33^, ^35^, ^37^, ^38^, ^39^, ^40^, ^41^
	People with asthma whose routine review is conducted remotely…	…via video consultation with document sharing/recording functions…	…are able to understand and engage in personalized discussions and information regarding their asthma condition and management.^27^, ^31^, ^33^, ^40^
	People with asthma whose routine review is conducted remotely…	…via telephone consultation…	…are able to understand and engage in personalized discussions and information regarding their asthma condition and management.^35^, ^36^, ^37^, ^39^
3. Provision of convenient/flexible access to advice and support	For people with asthma…	…the availability of HCPs to conduct remote consultations (video/telephone consultation)…	…can provide patients with a timely and appropriate option to gain advice and support from HCPs regarding their condition.^14^, ^17^, ^27^, ^29^, ^30^, ^33^, ^34^, ^39^
	HCPs conducting routine asthma reviews…	…when delivering review via remote consultation…	…can provide more convenient delivery of routine care for patients to access advice and support.^12^, ^27^, ^29^, ^35^
	People with asthma…	…when a routine review is conducted via video consultation or telephone…	…may find the mode of consultation delivery more convenient and flexible to fit their everyday lives, resulting in increased and flexible access to advice and support.^34^, ^39^
4. Enhanced healthcare professional‐patient relationships and communication with patients	People with asthma, scheduled for a routine asthma review…	…when a review is conducted with the same HCP each time…	…experience positive working relationships, which can be created and sustained, leading to positive patient outcomes.^17^, ^28^, ^30^, ^31^, ^33^, ^34^, ^37^, ^40^
	When an HCP is conducting a routine remote asthma review…	…and there is an existing relationship between patient and professional…	…the professional is able to engage the patient in shared decision‐making and self‐management strategies.^28^, ^30^, ^31^, ^34^, ^37^, ^40^
	When an asthma patient's review is conducted remotely…	…and via video consultation…	…collaborative discussions and self‐management strategies can be effectively communicated and discussed.^30^, ^40^
5. Appropriate provision of specific practical asthma self‐management strategies (action plans and inhaler technique)	People with asthma who are scheduled for a routine asthma review…	…that is conducted via telephone/video call, and includes a discussion/provision of a personalized asthma action plan…	…can experience increased understanding, enabling them to stay in control of their asthma, recognize symptoms of deterioration and what actions to take.^27^, ^28^, ^31^, ^34^, ^37^, ^38^, ^40^
	HCPs conducting a routine asthma review…	…when the review is conducted via remote consultation…	…can effectively communicate practical self‐management advice (e.g., inhaler technique and action plans) and enable collaborative discussions with patients.^27^, ^39^
	People with asthma whose review is conducted via video consultation…	…when provided with information on practical self‐management strategies (asthma action plans/inhaler technique)…	…is able to understand and engage in discussions regarding the best use of these tools.^27^, ^39^, ^40^
	People with asthma whose review is conducted via telephone consultation…	…when provided with information on practical self‐management strategies…	…is able to understand and engage in discussions regarding the best practice of using these tools and then HCP or patient is able to convert the information in written format.^31^, ^37^
6. Increased patient confidence & self‐efficacy	People with asthma who are scheduled for a routine asthma review…	…conducted via telephone or video consultation…	…can gain confidence to manage their own condition.^12^, ^27^, ^31^, ^33^, ^35^, ^36^, ^38^, ^39^, ^41^

#### 3.3.1 Theme 1: Increased regular attendance and increased monitoring of patient

*Patients*: For patients with asthma, the increase in regular attendance at reviews conducted remotely was due to a number of advantages, including increased convenience, time and cost savings for patients.^14^, ^17^, ^27^, ^29^, ^31^, ^33^ Remote reviews were perceived as better at meeting patient needs and preferences compared to a standard face‐to‐face review, as they reduced barriers to treatment and eased access to routine care.^14^, ^17^, ^27^, ^29^, ^31^, ^32^, ^34^, ^35^, ^36^, ^39^, ^40^ Regular attendance at remote reviews and supported self‐management delivery led to an increase in patient confidence and enablement in their asthma care.^33^, ^36^

*Professionals*: Symptoms could be monitored, reviewed, interpreted and acted on safely in remote consultations. Increased patient attendance at routine remote reviews created regular opportunities for healthcare professionals to provide feedback on monitored asthma symptoms to patients (e.g., monitoring peak flows and asthma triggers).^27^, ^28^, ^30^, ^34^, ^38^, ^39^ Additionally, the opportunity to maintain contact and ongoing monitoring was one of the most commonly recognized advantages of remote consultations.^27^, ^28^, ^34^ Patients' medication and asthma action plans could be reviewed, reinforcing earlier detection of symptoms or deterioration and timely self‐management.^38^, ^39^

*Video consultations*: In addition to enabling feedback on monitored asthma symptoms or behaviours, video consultations had particular advantages for monitoring a patient's condition through systems, such as ‘document camera’ or ‘picture‐in‐picture’ functions, which facilitated patients and professionals reviewing the contents of documents (e.g., asthma action plans) together.^27^, ^30^

*Telephone consultations*: A number of articles supported telephone reviews as an efficient way of maintaining contact with asthma patients.^34^, ^39^ Telephone consultations facilitated regular discussions and met patients' needs and preferences due to increased convenience, facilitating attendance at routine telephone reviews.

#### 3.3.2 Theme 2: Opportunities to provide individualized information about asthma and asthma management

*Patient*: Video and telephone consultations were a safe and effective mechanism to facilitate the delivery of individualized information about asthma and its management, resulting in increased patient understanding of their condition^27^, ^31^, ^33^, ^35^, ^37^, ^38^, ^39^, ^40^, ^41^ and improved overall asthma control.^37^ Remote consulting provided opportunities for patients to learn about their condition,^32^, ^36^ and increased patient satisfaction with the mode of consultation.^27^, ^31^, ^33^, ^40^

*Professional*: Use of video and telephone consultations were both recognized as effective communication strategies for healthcare professionals to provide individualized information, instructions, education and signposting of other essential resources to patients.^27^, ^31^, ^32^, ^33^, ^35^, ^37^, ^38^, ^39^, ^40^, ^41^

*Video consultations*: Patients found video consultation technology visually appealing and engaging, enhancing understanding and asthma education (e.g., information about asthma triggers).^27^, ^31^, ^33^, ^40^ Use of video technology‐facilitated greater discussion between patients and professionals.^27^, ^31^, ^33^, ^40^ Recording functions allowed patients to record their review then rewatch, consolidate and confirm the information discussed.^40^

*Telephone consultations*: Several studies supported the use of telephone consultations as an effective tool to deliver individualized information to patients.^35^, ^36^, ^37^ More specifically, telephone reviews were recognized as a timely,^39^ effective and efficient means to provide information and transfer instructions to patients to manage their asthma.

#### 3.3.3 Theme 3: Provision of convenient/flexible access to advice and support

*Patients*: Remote consultations provided more convenient and flexible access to advice and support for patients with asthma, compared to attending a face‐to‐face review.^34^, ^39^ Particular groups who favoured the convenience and timeliness of remote consultations were patients who lived in rural communities,^27^, ^30^ patients whose lives were structured around work, study or childcare,^17^ younger patients who were more familiar with the use of technology^29^, ^33^ and older, vulnerable patients with reduced mobility.^14^, ^33^ Ease of access was particularly helpful for patients who noticed a change in symptoms or peak flow readings and were able to contact a healthcare professional promptly via remote consultation.^29^ Remote asthma consultations may potentially narrow socioeconomic inequalities in access to healthcare, by being more accessible to vulnerable groups.^14^, ^33^

*Professionals*: Healthcare professionals may have more availability to conduct a remote video or telephone review, enabling them to respond more promptly than a face‐to‐face appointment may have offered.^12^, ^27^, ^29^, ^35^

*Video and telephone consultations*: For some patients, telephone and video consultations were a preferred method of consultation, and patients were more likely to attend this type of review, leading to increased engagement.^34^, ^39^

#### 3.3.4 Theme 4: Enhanced healthcare professional–patient relationships and communication

*Patients*: Patients whose reviews were conducted with the same clinician each time (potentially facilitated by remote consultations), reported better health‐related outcomes and greater satisfaction with the consultation.^28^, ^30^, ^31^, ^34^, ^37^, ^40^ Benefits described included increased shared decision‐making,^17^, ^33^, ^40^ more discussion of personal preferences^17^, ^33^ and increased attendance at reviews.^30^ Reviews conducted with the same clinician were seen to be particularly important to young people,^33^ leading to more engagement and increased confidence in self‐management strategies. The mechanism for this was the trust built during an existing relationship between professional and patient^.^ ^33^

*Professionals*: A number of studies suggested that when a relationship is already established between patient and professional, telephone and video technologies are a suitable platform to engage in shared decision‐making and discussion of self‐management strategies. The existing relationship ensures the professional recognizes changes in a person's condition due to their prior awareness of personal circumstances.^28^, ^30^, ^31^, ^34^, ^37^, ^40^

*Video consultation*: Patients were able to discuss their asthma action plan with their health professional during remote reviews. Video‐facilitated collaboration through technologies, such as ‘screening sharing’ and ‘editing documents’, allowed the patient and professional to work together to personalize their action plan.^30^, ^40^ Recording functions enabled patients to revisit their review and help consolidate the information delivered, to improve understanding of their asthma and how to manage their condition.^30^, ^40^

#### 3.3.5 Theme 5: Appropriate provision of specific practical asthma self‐management strategies (action plans and inhaler technique)

*Patients*: Specific practical asthma self‐management strategies can be effectively communicated, delivered and discussed during remote asthma reviews. An individualized, written asthma action plan can be successfully discussed via telephone or video consultation. Remote provision/discussion of an action plan leads to positive patient outcomes, such as increased patient understanding,^27^, ^28^, ^31^, ^34^, ^37^, ^38^, ^40^ improved control of their condition,^27^, ^31^, ^34^, ^37^, ^38^ increased quality of life,^28^ greater patient self‐efficacy^27^ and allows patients who may not regularly attend face‐to‐face reviews to have their action plan reviewed.^34^

*Professionals*: The use of video and telephone consultations is an effective alternative for discussing a patient's asthma plan compared to face‐to‐face reviews. Discussion of individualized action plan information and medications can be safely reviewed to increase patient understanding of their condition, medication adherence and how to recognize symptom deterioration. Professionals were able to demonstrate inhaler technique and provide education using the visual aids and tools of video consultation technologies effectively and safely.^27^, ^39^

*Video consultation*: When professionals are communicating and demonstrating practical strategies, such as inhaler technique via video consultation, patients were able to understand and learn from the instructions when the professional's video camera was positioned from the waist up (allowing the demonstration to be fully visualized).^27^ Similarly, healthcare professionals could review patients' technique. Online screen‐sharing technologies allowed patients and professionals to collaboratively edit asthma action plans during video consultations.^40^ This led to improved communication and avoided misunderstandings, and enhanced shared decision‐making between the individual and professional. The improved attendance at remote consultations enabled these specific skills to be reviewed with more patients.^39^

*Telephone consultation*: Telephone consultations are a safe and effective alternative to face‐to‐face reviews to discuss and provide practical self‐management advice and support. Individual asthma action plans can be discussed over the telephone and then converted into written versions and sent to patients after the consultation. This technique of discussions and provision of action plans were seen to significantly improve asthma control.^31^, ^37^

#### 3.3.6 Theme 6: Increased patient confidence and self‐efficacy

*Patient*: Through the increased engagement with a remote consultation and prompt clinical input, patients felt more empowered and had up‐to‐date strategies to manage their condition.^27^, ^31^, ^33^, ^35^, ^36^, ^38^, ^39^, ^41^ Patients also gained confidence to undertake self‐management techniques from regularly attending remote reviews, which they may have missed from nonattendance at a face‐to‐face review. Overall, this led to increased confidence in their understanding of how to identify impending attacks and in their ability to act appropriately.^12^, ^38^

### 3.4 Overarching synthesis

The overarching synthesis from the six key themes identified that, in relation to the review's key aims (to explore the safety, clinical effectiveness and safety of supported self‐management delivery in remote asthma consultations), remote consultations were overall, more highly accepted than in‐person consultations by many patients and professionals, and were an equally safe and effective alternative to face‐to‐face reviews. In only one instance were concerns raised about remote consulting,^29^ in particular with regard to clinical effectiveness and safety. Specifically, uncertainties about the effectiveness and quality of interactions compared to face‐to‐face meetings were raised. One further study^28^ suggested there was no perceived improvement of control where telemedicine alone was received, although this is not suggestive of poorer results. An overview of all findings has been presented in Table 3.

**Table 3:** Synthesis of findings corresponding to key aims

	Remote (video/telephone) versus face‐to‐face (in person) asthma consultations
Acceptability	(On average) higher levels of acceptability from both patients and professionals for remote delivery of asthma care
Safety	Remote consultations were recognized as safe as providing a face‐to‐face review
Clinical effectiveness	Remote consultations were recognized as clinically effective as providing a face‐to‐face review

## 4 DISCUSSION

### 4.1 Summary of findings

We identified six themes using data from 18 articles to describe how supported self‐management is delivered during remote asthma consultations. We identified positive benefits associated with remote asthma care, including increased convenience, improved access (including for some vulnerable groups) and attendance at reviews, ability to assess the core content of asthma remotely (especially video reviews that enabled practical tasks such as checking inhaler technique), completion of asthma action plans (screen sharing or discussed with documents sent postconsultation) and continuity of care. Typically, these overrode any challenges associated with distance imposed by remote consultations, and patient's concerns about the quality of the interaction. Overall, our data suggest that for many patients and healthcare professionals, remote consultations are more highly accepted than in‐person consultations, and were equally as effective and safe as face‐to‐face reviews.

### 4.2 Interpretation of findings of this study in relation to current literature

Guidelines for asthma management^2^, ^3^ recommend that asthma should be monitored (often in primary care) by routine clinical review on at least an annual basis. Every asthma consultation is an opportunity to review, reinforce and extend patient's knowledge and skills.^5^ Regular professional review is a core component of supported self‐management,^26^ with evidence of greater reductions in hospitalisations and emergency department visits in trials where the intervention includes regular review.^43^, ^44^ The findings of this realist review show that using remote means to provide consultations can increase patient engagement and attendance at asthma reviews.^14^, ^17^, ^27^, ^29^, ^30^, ^31^, ^32^, ^33^, ^34^, ^36^, ^39^ Our realist synthesis suggests that one mechanism for the benefits of telehealth communications is the convenience of telephone or video consultations, which facilitates attendance at reviews.^14^, ^17^, ^27^, ^29^, ^30^, ^33^, ^34^, ^39^

Providing patients with information and guidance for self‐management of their asthma is an essential aspect of all routine reviews. Our findings highlight that the use of telephone and video consultations is an acceptable, effective and safe alternative to face‐to‐face consultations for providing patients with this information. Importantly, the partnership between the patient and professional should enable information to be discussed, understood and agreed upon between both the patient and professional. Such ‘shared decision‐making’ can improve clinical outcomes and quality of life by actively engaging them in managing their own health.^45^ We found that telephone and video consultations have the potential to be effective platforms that can facilitate shared decision‐making.

Asthma is a variable condition and some people with asthma may be well controlled and need very little support for many months. However, when symptoms are triggered, access to professional care needs to be flexible in timing and mode of delivery.^46^ As an alternative to face‐to‐face consultations, the findings of this study highlight that remote asthma reviews can provide flexible and convenient access to professional support enabling patients to be provided with appropriate and prompt clinical input. Such flexible access to their healthcare professionals promotes patients' confidence in their ability to self‐manage their condition.^12^, ^27^, ^31^, ^33^, ^35^, ^36^, ^38^, ^39^, ^41^

The provision of a personalized asthma action plan is an essential strategy in supporting people with asthma to take the right actions at the right time.^2^, ^3^, ^6^, ^47^ People with asthma spend a matter of minutes in a routine review with their healthcare professional; the rest of the time, they are making their own decisions about their medications and when they should seek medical help. It is therefore essential that asthma reviews are used to agree on what they should do if their asthma control deteriorates and to empower them to take timely and appropriate action. Findings from this review highlight the acceptability, clinical effectiveness and safety of delivering action plans in remote routine reviews.

Kew and Cates^34^ in a Cochrane review concluded that there were no important differences between face‐to‐face and remote asthma reviews in terms of exacerbations, asthma control or quality of life, though there was insufficient information to rule out differences in efficacy or safety. Consistent with the ‘what/how/context’ aims of a realist synthesis,^18^, ^19^, ^20^, ^21^ our findings extend the Cochrane review by identifying which aspects of supported self‐management can be delivered via remote means, describing strategies that enable the provision of video‐ or telephone consultations, and for whom and under what circumstances remote reviews may be most beneficial.

Kearney^48^ reflects on the fast pace at which UK NHS services have moved to remote care when the COVID‐19 pandemic demanded social distancing, concluding that it will be essential for future healthcare services to *‘* do things differently’ in their approach to LTCs and the delivery of supported self‐management. The report concludes that it is critical to plan carefully for the use of remote technologies and to identify the best practice of self‐management delivery at scale and in a sustainable way. The findings of our review provide the context and mechanisms for effective remote asthma‐supported self‐management delivery.

### 4.3 Strengths and limitations

To our knowledge, we have conducted the first rapid realist review in the area of asthma supported self‐management delivery via remote consultation. Our review is timely given the shift to remote care driven by the COVID‐19 pandemic, and we explored the (rapidly expanding) use of video and/or telephone consultations, and systematically identified the perceived benefits and challenges of each mode of delivery in relation to each theme. Additionally, the findings are explored through the perspective of both the health professional and the patient. A final strength is that we utilized a robust realist methodology, which is gaining recognition for its contribution to healthcare research.^49^

A weakness of our study was the time constraints that we overcame by the use of a rapid realist review. By design, our review was ‘rapid’, and we recognize that a more detailed approach such as a traditional realist synthesis may have revealed, challenged or confirmed some of the themes presented in the findings of this study, due to its ability to test presented theories. However, we believe our findings have been systematically constructed, and all feedback provided by the multidisciplinary External Reference Group was considered and actioned. Additionally, the use of the PRISMS taxonomy^26^ as a framework for analysis allowed the structure and interpretation to be grounded in the existing evidence base. A weakness of realist methodology is the subjectivity of the data extraction and the challenge of extracting unbiased C–M–Os. The primary research studies are generally not reported in line with realist concepts (C–M–O configurations) and therefore data extraction requires the researcher to interpret data to explore the context and mechanistic features of the research. In addition, we also acknowledge some limitations in the interpretation of the findings. Although every effort was made to ensure a nonbiased approach to data extraction, we recognize that the included studies may be liable to publication bias with a focus on more successful components of their interventions, and favour reporting of positive or significant findings, resulting in an overly positive interpretation of the effects of remote consulting. To address this, we ensured our data extraction included all intervention outcomes (successful or not), and specifically highlighted where fewer positive findings were noted, although these were infrequent and insufficient to form a theme. For example, in Godden and King^29^ some professionals expressed concerns about the quality of remote consultations considering that they may not be as effective as face‐to‐face reviews. They described varied opinions on communicating key information remotely, as well as concerns about patient's willingness to accept new technologies. Although these negative opinions, were outweighed by the potential advantages of remote consultations in empowering people to manage their condition and enabling timely management of exacerbations, in the studies included in this review, this study does however raise the point that patient preference is always important to consider.

To increase the reliability of findings, we involved a second reviewer in the data extraction phase. The research team regularly discussed potential findings to ensure different perspectives were considered and resulted in a balanced interpretation of the data. The aim was to reach a consensus in interpretation, and this was achieved for all findings.

This review was completed during the COVID‐19 pandemic period, but all studies included in the data predated the pandemic. Post‐COVID research may present different findings as healthcare adapts to new models of asthma care. Additionally, we were dependent on the completeness of the included studies, so some potentially important contexts may not have been evaluated. For example, we did not have evidence to inform the role of remote support for self‐management in the context of people living with disabilities, or ethnic minority groups potentially with language barriers. Future research should specifically explore remote supported self‐management delivery for such groups.

We also acknowledge that although the review findings indicate either equivalence or greater benefit of remote self‐management delivery, there will be individuals for whom face‐to‐face reviews are a preferred mode of healthcare delivery and communication. Additionally, we recognize that the ‘safety’ variable measured within this review has not been tested within a controlled trial. Although we found no indication that remote delivery of supported self‐management caused harm, we would recommend future studies to explore this further.

### 4.4 Implications for future research and policy

Future research should explore how telecommunication can be implemented in ways that are most valued by patients and clinicians, to fit within the organisational and technical infrastructure of healthcare services and embrace the culture of delivering supported self‐management.^33^ Asthma UK^50^ advocate that policy makers and innovators need to work together to develop a national effort towards delivering sustainable supported self‐management and long‐term implementation of improved patient‐centred asthma care. The Asthma UK Centre for Applied Research (AUKCAR) is a collaborative network of applied asthma researchers, clinical and academic respiratory experts, as well as PhD students and asthma patient representatives.^50^ Supported self‐management is a key theme within the AUKCAR and the IMP^2^ART programme of work has developed evidence‐based, practical strategies to promote the delivery of supported self‐management in routine primary care.^51^, ^52^, ^53^ This rapid realist review provides evidence‐based findings of the underlying contexts and mechanisms in remote service provision that contribute towards effective supported self‐management delivery during asthma reviews, which will be highlighted by the IMP^2^ART programme.

## 5 CONCLUSIONS

Even when the COVID‐19 pandemic recedes, remote technologies will remain in everyday healthcare. This paper highlights new knowledge through the use of realist methodology by understanding the existing mechanisms and the interplay within differing contexts, and has revealed how and why remote supported self‐management for asthma can be effectively delivered. A core component of asthma care is supporting self‐management, a guideline‐recommended intervention that reduces the risk of acute attacks, and improves asthma control and quality of life. Across a broad range of contexts, remote consultations are highly accepted by both patients and professionals, and are as clinically effective and safe as face‐to‐face reviews to provide self‐management support. Specific groups advantaged by remote consulting included those living in rural communities, or who had to fit their healthcare around work or domestic responsibilities, and those with reduced mobility. The findings of this rapid realist review can inform the conduct of remote asthma reviews, and implementation of supported self‐management techniques into asthma care.

## CONFLICT OF INTERESTS

The authors declare that there are no conflict of interests.

## AUTHOR CONTRIBUTIONS

Emma Kinley undertook the data collection, extraction and synthesis. Imogen Skene independently screened the data search and extracted 25% of the data to ensure reliability and validity. All authors (Emma Kinley, Imogen Skene, Elizabeth Steed, Kirstie McClatchey, Hilary Pinnock) contributed to data interpretation and critically revised the manuscript. All authors read and approved the final manuscript.

## Supporting information

Supplementary information.

Supplementary information.

Supplementary information.

## Data Availability

All data generated or analysed during this study are included in this published article (and its Supporting Information Files).
