# Supplementary material for: Delivery of supported self‐management in remote asthma reviews: A systematic rapid realist review
Source: Health Expect. 2022 Apr 11;25(4):1200–14. doi: 10.1111/hex.13441 (PMC9327809; doi:10.1111/hex.13441)
Supplement: Supplementary file 1 — Supplementary information. [file HEX-25--s002.docx]

| 1) Database: **Ovid MEDLINE(R)**1946 to September Week 4 2020 | | |
| --- | --- | --- |
| **#** | **Searches** | **Results** |
| 1 | Asthma, Aspirin-Induced/ or Asthma, Occupational/ or Asthma/ or Asthma, Exercise-Induced/ or Asthma-Chronic Obstructive Pulmonary Disease Overlap Syndrome/ or asthma.mp. | 161522 |
| 2 | telecommunications/ or telemedicine/ or remote consultation/ or telephone/ or videoconferencing/ or "health care (non mesh)"/ | 43517 |
| 3 | 1 and 2 | 369 |
| 2) Database: **Embase**1980 to 2020 Week 40 | | |
| **#** | **Searches** | **Results** |
| 1 | experimental asthma/ or moderate persistent asthma/ or extrinsic asthma/ or mild intermittent asthma/ or asthma-chronic obstructive pulmonary disease overlap syndrome/ or nocturnal asthma/ or severe persistent asthma/ or thunderstorm asthma/ or intrinsic asthma/ or asthma/ or allergic asthma/ or asthma.mp. or mild persistent asthma/ or occupational asthma/ or exercise induced asthma/ | 281162 |
| 2 | telecommunication/ or teleconference/ or teleconsultation/ or telemedicine/ or telephone interview/ or telehealth/ | 78477 |
| 3 | 1 and 2 | 953 |
| 3) Database: **APA PsycINFO**1987 to September Week 4 2020 | | |
| **#** | **Searches** | **Results** |
| 1 | asthma/ | 4184 |
| 2 | exp Professional Consultation/ or exp Videoconferencing/ or exp Telemedicine/ or exp Technology/ | 219084 |
| 3 | 1 and 2 | 138 |
| 4) Database: Cochrane Library, Date Run: 05/10/2020 16:17:50 | | |
| **#** | **Searches** | **Results** |
| 1 | (asthma):ti,ab,kw (Word variations have been searched) with Publication Year from 2000 to 2020, in Trials | 22372 |
| 2 | remote consultation with Publication Year from 2000 to 2020, in Trials | 513 |
| 3 | telemedicine with Publication Year from 2000 to 2020, in Trials | 3857 |
| 4 | teleconference with Publication Year from 2000 to 2020, in Trials | 163 |
| 5 | video conference with Publication Year from 2000 to 2020, in Trials | 2417 |
| 6 | remote care with Publication Year from 2000 to 2020, in Trials | 2072 |
| 7 | telecare with Publication Year from 2000 to 2020, in Trials | 413 |
| 8 | telephone with Publication Year from 2000 to 2020, in Trials | 17866 |
| 9 | #2 OR #3 OR #4 OR #5 OR #6 OR #7 OR #8 | 24847 |
| 10 | #1 AND #9 | 506 |
