# Supplementary material for: Delivery of supported self‐management in remote asthma reviews: A systematic rapid realist review
Source: Health Expect. 2022 Apr 11;25(4):1200–14. doi: 10.1111/hex.13441 (PMC9327809; doi:10.1111/hex.13441)
Supplement: Supplementary file 2 — Supplementary information. [file HEX-25--s001.docx]

| **Reference** | **Study Design** | **Study Location** | **No. of Participants / Studies** | **Mode of Remote Delivery** | **Outcomes Assessed** | **PRISMS Components of CMOs** | **Key Findings/ Outcomes (of CMO configurations)** | **Relevance to Safety, Clinical Effectiveness & Acceptability** |
| --- | --- | --- | --- | --- | --- | --- | --- | --- |
| Brown W, Schmitz T, Scott DM, Friesner D. Is Telehealth Right for Your Practice and Your Patients With Asthma? *Journal of Patient Experience*. 2017;4(1):46-49. doi:10.1177/2374373516685952 | Cohort Pilot Study | United States of America | 18 asthma Patients | Telehealth with real-time communication (telephone & video consultations) | Patient satisfaction with telemedicine experience survey | A1, A3, A4, A5, A11 | Telehealth resources are an acceptable and convenient means to deliver asthma education and regular routine reviews for patients in a rural, medically underserved community.  Through use of graphics and pictures during the video consultation, patients facilitated greater discussion and perceived retention of information about common asthma triggers.  Written asthma action plans provided via telehealth can help increase patient self-efficacy to use their action plans. This study acknowledged patients were able to recognised their early warning symptoms and review details of their action plans, resulting in taking relief medication. Professionals are able to review documents provided by patients e.g., asthma symptom diary, and are able to provide feedback to the patient via remote technologies. Video consultations facilitated demonstration of inhaler technique when the camera was positioned from the waist up. | Acceptability & Clinical Effectiveness |
| Chongmelaxme B, Lee S, Dhippayom T, Saokaew S, Chaiyakunapruk N, Dilokthornsakul P. The Effects of Telemedicine on Asthma Control and Patients’ Quality of Life in Adults: A Systematic Review and Meta-analysis. *The Journal of Allergy and Clinical Immunology: In Practice*. 2019;7(1):199-216.e11. doi:10.1016/j.jaip.2018.07.015 | Systematic Review and Meta-Analysis | United States of America, United Kingdom, Asia, Europe | 22 studies | Tele-consultations & tele-case management | Asthma Control and Asthma Patient's Quality of life | A3, A5 | Improvements in patient quality of life were found when tele-case management was used, in comparison to all other tele-medicine approaches. The tele-case management study included: collaborative patient’s self-management, monitoring patient’s health status, interactive communication and provision of an action plan. | Clinical Effectiveness |
| Donaghy E, Atherton H, Hammersley V, et al. Acceptability, benefits, and challenges of video consulting: a qualitative study in primary care. *British Journal of General Practice*. 2019;69(686):e586-e594. doi:10.3399/bjgp19x704141 | Qualitative Study (semi-structured interviews with patients and clinicians) | United Kingdom | 21 asthma patients and 13 clinicians | Video consultations | Patients and clinicians’ experiences of video consultations | A4, A8, A9 | Opportunities for remote reviews resulted in increased patient attendance to their regular review (easier access to advice and support).  Continued, existing doctor-patient relationships facilitated greater communication and shared decision making between patient and healthcare professionals for self-management decisions. | Acceptability & Clinical Effectiveness |
| Godden DJ, King G. Rational development of telehealth to support primary care respiratory medicine: patient distribution and organisational factors. *Primary Care Respiratory Journal*. 2011;20(4):415-420. doi:10.4104/pcrj.2011.00063 | Qualitative Study (semi-structured interviews with clinicians) | United Kingdom | 20 clinicians | Telehealth | Clinician perspectives of telehealth | A8, A4 | Interacting with patients via different technologies, which they are comfortable with, can work more conveniently for patients, and they are therefore more likely to attend their routine review.  Telemonitoring and conducting routine reviews (via telephone or video), can result in people with asthma being more proactive with their self-management, as they are able to contact a healthcare professional for a more convenient and timely review (identification of early exacerbations). | Acceptability & Safety |
| Goodridge D, Marciniuk D. Rural and remote care. *Chronic Respiratory Disease*. 2016;13(2):192-203. doi:10.1177/1479972316633414 | Literature Review | Canada | Not applicable | Telehealth | Explores implementation of telehealth in rural and remote settings | A5, A6, A7 | Remote technologies can promote patient-centred care by facilitating communication between patients and supporting self-management with provider feedback.  The American Medical Association supports prescribing medicine over remote consultations, provided that the patient and healthcare professional have an existing relationship. | Acceptability, Clinical Effectiveness & Safety |
| Greenhalgh T, Shaw S, Wherton J, et al. Real-World Implementation of Video Outpatient Consultations at Macro, Meso, and Micro Levels: Mixed-Method Study. Journal of Medical Internet Research. 2018;20(4):e150. doi:10.2196/jmir.9897 | Mixed Methods Study | United Kingdom | 24 clinician interviews, 30 videotaped remote consultations and 17 audiotaped face-to-face consultations | Video consultations | Good practice and implementation of video consultations | A4, A8 | Patients showed greater engagement, improved self-management, overall control, and a significant reduction in ‘did not attend’ rates when attending remote consultations.  Remote consultations allows prompt clinical input, and improved patient confidence in self-management. | Acceptability & Clinical Effectiveness |
| Gruffydd-Jones K, Hollinghurst S, Ward S, Taylor G. Targeted routine asthma care in general practice using telephone triage. British Journal of General Practice. 2005;55(521):918-23. | RCT | United Kingdom | 194 asthma patients | Telephone consultations | Asthma Control, NHS costs, Quality of life, Exacerbations | A1, A3, A4 | Remote consultations significantly improve access to routine care. Patients are more likely to receive their annual review if conducted via telephone.  Increased patient understanding of individual condition, shared decision making between patient and professional, and provision of action plan. | Acceptability & Clinical Effectiveness |
| Hanlon P, Daines L, Campbell C, McKinstry B, Weller D, Pinnock H. Telehealth Interventions to Support Self-Management of Long-Term Conditions: A Systematic Metareview of Diabetes, Heart Failure, Asthma, Chronic Obstructive Pulmonary Disease, and Cancer. Journal of Medical Internet Research. 2017;19(5). doi:10.2196/jmir.6688 | Meta-Review | United States of America, Europe, Asia, Oceania | 53 systematic reviews (232 RCTs) | Telehealth | Effective self-management support | A1, A3, A5 | There are little or no significant differences in the provision of supported self-management components (PRISMS) between remote and face-to-face care for asthma (different for other long-term conditions). Remote care is a safe alternative mode of delivery of self-management support (meta-analysis results). | Clinical Effectiveness |
| Ignatowicz A, Atherton H, Bernstein CJ, et al. Internet videoconferencing for patient–clinician consultations in long-term conditions: A review of reviews and applications in line with guidelines and recommendations. Digital Health. 2019;5. doi:10.1177/2055207619845831 | Review of Reviews | Not stated | 35 review articles (systematic, meta-review and literature reviews) | Video conferencing (only asthma outcomes were assessed) | Summarises existing reviews use of internet video conferencing and patients with long-term conditions (patient satisfaction/ patient outcomes) | A1, A4, A8, A9 | Implementation of videoconferences for routine asthma reviews can reduce barriers to treatment and increase convenience for patients.  Improved relationship between patient and professional, which lead to more frequent contact with the specific clinician who is known to the patient and likely to know particular young patient’s personal circumstances and what is important to them.  Provision of patient education during routine video consultations can increase patient satisfaction and improve health outcomes. | Acceptability & Clinical Effectiveness |
| Kew KM, Cates CJ. Home telemonitoring and remote feedback between clinic visits for asthma. Cochrane Database of Systematic Reviews. Published online August 3, 2016. doi:10.1002/14651858.cd011714.pub2 | Systematic Review | United Kingdom, United States of America, Netherlands, Denmark | 6 studies including 2100 participants | Remote consultations using technology (telephone, video consultations). | Safety and efficacy of remote vs face-to-face asthma consultations | A3, A4, A5, A6 | Remote asthma consultations provides an unobtrusive and efficient way of maintaining contact with patients. Remote check-ups may not disrupt a person’s life in the way a regular clinic visit might and may serve to enhance self-management behaviours such as keeping a personalised action plan up to date and adherence to medications. | Acceptability & Clinical Effectiveness |
| Pinnock H. It’s good to talk… … but do I really need to see you? The potential of telephone consultations for providing routine asthma care. Primary Care Respiratory Journal. 2003;12(3):79-80. doi:10.1038/pcrj.2003.50 | Editorial Response | United Kingdom | Not applicable | Telephone consultations vs face-to-face | The potential of telephone consultations for providing routine asthma care | A1, A2, A4, A8, A14 | Telephone reviews help overcome the barrier of access to care which may otherwise take up a lot of patient’s time to access a clinic.  Patients can be provided with information regarding their asthma and management of their asthma, are able to be signposted to supporting literature/websites for available resources and can be provided with advice and support around health and lifestyle e.g., stopping smoking, via telephone consultation. | Acceptability, Clinical Effectiveness & Safety |
| Pinnock H, Adlem L, Gaskin S, Harris J, Snellgrove C, Sheikh A. Accessibility, clinical effectiveness, and practice costs of providing a telephone option for routine asthma reviews: phase IV controlled implementation study. British Journal of General Practice. 2007;57(542):714-22. | Before- and after implementation study | United Kingdom | 1809 asthma patients in 1 UK practice | Telephone consultations vs face-to-face | Uptake of telephone reviews (impact on review rates), asthma morbidity and cost to practice | A1, A4 | Patients provided with a routine telephone review offer a stable ‘maintenance’ phase of monitoring, during which self-management assumes precedence. In turn, can increase patient’s confidence in managing their own condition. | Clinical Effectiveness |
| Raju JD, Soni A, Aziz N, Tiemstra JD, Hasnain M. A patient-centered telephone intervention using the asthma action plan. Family medicine. 2012;44(5):348-50. | Prospective cohort study | United Kingdom | 48 adult patients with asthma | Telephone consultations vs face-to-face | Demonstrate the utility of action plan implementation by phone to improve asthma control | A1, A3, A4 | Asthma control can not only be assessed via telephone, but also significantly improved when the action plan is discussed with the healthcare professional during routine reviews.  Asthma control can not only be assessed via telephone, but also significantly improved when the action plan is discussed with the healthcare professional during routine reviews. | Acceptability & Clinical Effectiveness |
| Van Gaalen JL, Hashimoto S, Sont JK. Telemanagement in asthma. Current Opinion in Allergy & Clinical Immunology. 2012;12(3):235-240. doi:10.1097/aci.0b013e328353370 | Review | United Kingdom | Not applicable | Tele-management for asthma | Developments in tele-management for the management of people with asthma | A1, A3, A5, A8 | Use of telemedicine provides patients with the tools to self-manage and gain control over their condition (self-monitoring, patient is able to detect and respond to symptom worsening and can easily contact a professional).  Use of telemedicine enables proactive individual patient care through the provision of a personalised asthma action plan. | Clinical Effectiveness |
| Vitacca M, Comini L, Scalvini S. Is teleassistance for respiratory care valuable? Considering the case for a “virtual hospital.” Expert Review of Respiratory Medicine. 2010;4(6):695-697. doi:10.1586/ers.10.75 | Editorial Response/ Expert Review | Italy | Not applicable | Teleassistance for respiratory care | Teleassistance in providing respiratory care | A1, A4, A5, A6, A11 | Results in quick transmission of information and clinical data in real-time, thus leading to greater continuity of care. Can provide active education and support. Use of monitoring via telemedicine can result in earlier detections of symptoms exacerbations. As these measures may be missed by a patient who would not visit a practice for a face-to-face review. | Clinical Effectiveness |
| Hamour O, Smyth E, Pinnock H. Completing asthma action plans by screen-sharing in video-consultations: practical insights from a feasibility assessment. npj Primary Care Respiratory Medicine. 2020;30(1). doi:10.1038/s41533-020-00206-8 (Identified by External Reference Group) | Feasibility Assessment | United Kingdom, Canada, Switzerland | 10 participants | Video consultations | Practicality, feasibility and utility of using screen-sharing technologies to complete asthma action plans remotely | A1, A3, A9 | Patients felt editing the document with the clinician collaboratively improved communication and avoided misunderstandings. It also enhanced shared decision making between the individual and professional.  Patients can revisit their review, and help consolidate the information delivered during to better understand their asthma and how to manage their condition.  Online screen-sharing is a practical approach to joint completion of asthma action plans. | Acceptability, Clinical Effectiveness & Safety |
| Paré G, Moqadem K, Pineau G, St-Hilaire C. Clinical Effects of Home Telemonitoring in the Context of Diabetes, Asthma, Heart Failure and Hypertension: A Systematic Review. *Journal of Medical Internet Research*. 2010;12(2):e21. doi:10.2196/jmir.135 (Identified by External Reference Group) | Systematic Review | United States of America, Europe, Asia | 62 studies | Telemonitoring/teleconsultation | Clinical effects associated with home telemonitoring programmes in the context of chronic diseases | A5, A9 | During remote consultations, patients are able to actively participate in their own care and as such, consultations may result in fewer asthma related symptoms, and improved overall asthma control. | Acceptability & Clinical Effectiveness |
| Thiyagarajan A, Grant C, Griffiths F, Atherton H. Exploring patients’ and clinicans’ experiences of video consultations in primary care: a systematic scoping review. BJGP Open. Published online March 17, 2020:bjgpopen20X101020. doi:10.3399/bjgpopen20x101020(Identified by External Reference Group) | Systematic Scoping Review | United Kingdom, United States of America | 7 studies | Video consultations | Patients and professionals’ experiences of video consultations in primary care | A8 | Remote consultation (specifically video consultations) can lead to improved access to support. Video consultations may be more convenient for patients, but is not considered superior to a face-to-face consultation. | Acceptability |
| *Only data relevant to the current review extracted.  ***Abbreviations:*** *CMOs = Context-Mechanism-Outcome Configuration; PRISMS = Practical Reviews In Self-Management Support; RCT = Randomised controlled trial.*  *PRISMS^[26]^ Components Explained:*  *A1. Information about condition and /or its management*  *A2. Information about available resources*  *A3. Provision of/agreement on specific clinical action plans and/or rescue medication*  *A4. Regular clinical review*  *A5. Monitoring of condition with feedback*  *A6. Practical support with adherence (medication or behavioural)*  *A7. Provision of equipment*  *A8. Provision of easy access to advice or support when needed*  *A9. Training/rehearsal to communicate with healthcare professionals*  *A10. Training/ rehearsal for everyday activities*  *A11. Training/ rehearsal for practical self-management activities*  *A12. Training/ rehearsal for psychological strategies*  *A13. Social support*  *A14. Lifestyle advice and support (Full definitions can be found within Table 5).* | | | | | | | | |
