# Supplementary material for: Delivery of supported self‐management in remote asthma reviews: A systematic rapid realist review
Source: Health Expect. 2022 Apr 11;25(4):1200–14. doi: 10.1111/hex.13441 (PMC9327809; doi:10.1111/hex.13441)
Supplement: Supplementary file 3 — Supplementary information. [file HEX-25--s003.docx]

| **No.** | **Reference** | **Context – Mechanism - Outcomes Configurations** | | | | Safety/ Clinical Effectiveness/ or Acceptability? | PRISMS Taxonomy Component? |
| --- | --- | --- | --- | --- | --- | --- | --- |
|  |  | Background information e.g., setting and demographics to outline possible **Contextual** factors; | Key workings that contributed to the design and functioning of a pathway to identify **Mechanisms** and resources; | | Information and evidence suggestive of the successes or failures of different aspects of an intervention (**Outcomes**) |  |  |
|  |  |  | Resource | Reaction/Response |  |  |  |
| **1** | Brown, W., T. Schmitz, D. M. Scott and D. Friesner (2017). "Is Telehealth Right for Your Practice and Your Patients With Asthma?" Journal of patient experience 4(1): 46-49. | Participants with asthma in a rural, medically underserved community | Introduction of telehealth technology (audio/video links) as a resource to conduct regular asthma reviews | Positive - Participants found the telehealth technology convenient | Telehealth resources are an acceptable and convenient means to deliver asthma education and regular routine reviews for patients in a rural, medically underserved community. | Acceptability | A1. Information about Condition and/or its management (asthma education)  A4. Regular Clinical Review |
|  |  | Participants with asthma in a rural, medically underserved community | Provision of written asthma action plan via telehealth technology (audio/video link) | Empowered self-efficacy of patients has a direct impact on health behaviour change | Written asthma action plans provided via telehealth can help increase patient self-efficacy to use their action plans. This study acknowledged patients were able to recognise their early warning symptoms and review details of their action plans, resulting in taking relief medication. | Clinical Effectiveness | A3. Provision of/agreement on specific clinical action plans and/or rescue medication |
|  |  | Participants with asthma in a rural, medically underserved community | Telehealth technology (video consultation) to review inhaler technique. (Camera should be positioned from waist up to recognise non-verbal cues) | Positive – increases patients’ comfort and understanding | Patients and professionals were clearly able to see demonstration of inhaler technique when camera is positioned from waist up. | Clinical Effectiveness | A11. Training/ Rehearsal for practical self-management (inhaler technique) |
|  |  | Participants with asthma in a rural, medically underserved community | Telehealth technology (video consultation) to review documents provided by patients (Additional advantages of the system such as ‘document camera’ or ‘picture-in-picture’ functions) | Positive – increased collaboration and understanding between patient and professional | Professional is able to review documents provided by patients e.g., asthma symptom diary, and able to provide feedback to the patient. | Clinical Effectiveness | A5. Monitoring of Condition with Feedback |
|  |  | Participants with asthma in a rural, medically underserved community | Telehealth technology (video consultation), use of graphics and pictures through video conferences to enhance instruction and understanding about asthma triggers | Positive – Patient finds the consultation more ‘visually appealing’ and engaging | Through use of graphics and pictures during the video consultation, patients facilitated greater discussion and perceived retention of information about common triggers. | Clinical Effectiveness | A1. Information about Condition and/or its management (asthma education) |

| **No.** | **Reference** | **Context – Mechanism - Outcomes Configurations** | | | | Safety/ Clinical Effectiveness/ or Acceptability? | PRISMS Taxonomy Component? |
| --- | --- | --- | --- | --- | --- | --- | --- |
|  |  | Background information e.g., setting and demographics to outline possible **Contextual** factors; | Key workings that contributed to the design and functioning of a pathway to identify **Mechanisms** and resources; | | Information and evidence suggestive of the successes or failures of different aspects of an intervention (**Outcomes**) |  |  |
|  |  |  | Resource | Reaction/Response |  |  |  |
| **2** | Chongmelaxme, B., S. Lee, T. Dhippayom, S. Saokaew, N. Chaiyakunapruk and P. Dilokthornsakul (2019). "The Effects of Telemedicine on Asthma Control and Patients' Quality of Life in Adults: A Systematic Review and Meta-analysis." The Journal of Allergy & Clinical Immunology in Practice 7(1): 199-216.e111. | Adults with asthma receiving a routine review | Use of ‘tele-case management’ approaches (healthcare professional interactions with patients) to conduct routine asthma review | Positive | This paper highlighted a significant improvement in patient quality of life when tele-case management was used, in comparison to all other tele-medicine approaches. The tele-case management study included: ‘collaborative patient’s self-management, monitoring patient’s health status, interactive communication and provision of an action plan’. (pg. 209) | Clinical Effectiveness | A5. Monitoring of condition with feedback  A3. Provision of/ agreement on specific clinical action plans and or/ rescue medication |
|  |  | Adults receiving telemedicine review for asthma | Telemedicine review for asthma control (combined telemedicine) | Improved asthma control, Improved quality of life | Results of this study also suggest that the duration of intervention  might affect the treatment effects of telemedicine. We found that the studies with telemedicine delivered for at least 6 months had higher proportions of patients with significant improvements  in asthma control and patients’ quality of life compared with the  studies with less than 6 months (p16) | Clinical Effectiveness | A1. Information about condition and /or its management  A3. Provision of/agreement on specific clinical action plans and/or rescue medication  A5. Monitoring of condition with feedback |
|  |  | Adults receiving telemedicine review for asthma | Telemedicine review for asthma control (single) | No improvement in control  Improved quality of life | Tele-case management was the only effective single telemedicine for the improvement of patients’  quality of life. We believe that this is because most tele-case  management incorporated several medical supportive managements, including a collaborative patients’ self-management, monitoring patients’ health status, interactive communication,  and an action plan provision. (p11) | Clinical Effectiveness/ Acceptability | A1. Information about condition and /or its management  A3. Provision of/agreement on specific clinical action plans and/or rescue medication  A5. Monitoring of condition with feedback |

| **No.** | **Reference** | **Context – Mechanism - Outcomes Configurations** | | | | Safety/ Clinical Effectiveness/ or Acceptability? | PRISMS Taxonomy Component? |
| --- | --- | --- | --- | --- | --- | --- | --- |
|  |  | Background information e.g., setting and demographics to outline possible **Contextual** factors; | Key workings that contributed to the design and functioning of a pathway to identify **Mechanisms** and resources; | | Information and evidence suggestive of the successes or failures of different aspects of an intervention (**Outcomes**) |  |  |
|  |  |  | Resource | Reaction/Response |  |  |  |
| **3** | Donaghy, E., H. Atherton, V. Hammersley, H. McNeilly, A. Bikker, L. Robbins, J. Campbell and B. McKinstry (2019). "Acceptability, benefits, and challenges of video consulting: a qualitative study in primary care." British Journal of General Practice 69(686): e586-e594. | For patients with a long-term condition, whose lives are structured around work, study or childcare | The opportunity to have a routine review conducted via remote consultation | Positive patient convenience, time and cost savings | Increased attendance for patients to their regular review (easier access to advice and support). | Acceptability | A8. Provision of easy access to advice and support when needed/ A4 Regular clinical Review |
|  |  | Patients with a long-term condition | Routine review for condition conducted via remote consultation with a professional they have an existing relationship with (or have had previous consultations with) | Positive, patient feels more comfortable, rapport is already existing | Improved doctor-patient relationships results in better communication and shared decision making between patient and healthcare professional for self-management decisions. | Acceptability / Clinical Effectiveness | A9. Training /rehearsal to communicate with healthcare professionals |

| **No.** | **Reference** | **Context – Mechanism - Outcomes Configurations** | | | | Safety/ Clinical Effectiveness/ or Acceptability? | PRISMS Taxonomy Component? |
| --- | --- | --- | --- | --- | --- | --- | --- |
|  |  | Background information e.g., setting and demographics to outline possible **Contextual** factors; | Key workings that contributed to the design and functioning of a pathway to identify **Mechanisms** and resources; | | Information and evidence suggestive of the successes or failures of different aspects of an intervention (**Outcomes**) |  |  |
|  |  |  | Resource | Reaction/Response |  |  |  |
| **4** | Godden, D. J. and G. King (2011). "Rational development of telehealth to support primary care respiratory medicine: patient distribution and organisational factors." Primary Care Respiratory Journal. 22. | Person with asthma measuring their peak flow and notice a difference in reading | Availability of HCP via video or telephone consultation to review symptoms | Seeking help from a professional | Telemonitoring and conducting routine reviews (via telephone or video), can result in people with asthma being more proactive with their self-management as they are able to contact a health professional for a more convenient and timely review (Identification of early exacerbations). | Safety | A8. Provision of easy access to advice or support when needed |
|  |  | A young person with asthma, who regularly uses technology | Routine asthma review conducted via remote consultation | Positive, comfortable with using the technology | Interacting with patients via different technologies which they are comfortable with, can work more conveniently for patients, and are therefore more likely to attend their routine review. | Acceptability | A4. Regular Clinical Review |
|  |  | Telehealth for adults | Telehealth appointment | Expediate appointments | The potential for telemonitoring to identify early exacerbations of disease was also noted | Safety/ Clinical Effectiveness | A5. Monitoring of condition with feedback  A1. Information about condition and /or its management |
|  |  | Telehealth for adults | Telehealth appointment | Professional response: Concern about quality of interaction vs face to face | For videoconferencing and remote monitoring there were uncertainties about effectiveness and the quality of interactions compared to face-to-face meetings or consultations that affected confidence, particularly with life threatening illness. | Safety/ Clinical Effectiveness |  |

| **No.** | **Reference** | **Context – Mechanism - Outcomes Configurations** | | | | Safety/ Clinical Effectiveness/ or Acceptability? | PRISMS Taxonomy Component? |
| --- | --- | --- | --- | --- | --- | --- | --- |
|  |  | Background information e.g., setting and demographics to outline possible **Contextual** factors; | Key workings that contributed to the design and functioning of a pathway to identify **Mechanisms** and resources; | | Information and evidence suggestive of the successes or failures of different aspects of an intervention (**Outcomes**) |  |  |
|  |  |  | Resource | Reaction/Response |  |  |  |
| **5** | Goodridge, D. and D. Marciniuk (2016). "Rural and remote care." Chronic Respiratory Disease 13(2): 192-203. | GP practices in rural and remote communities | Providing telehealth care (remote monitoring and remote consultations) for asthma patients | Positive patient outcomes (relationship with professional and increased supported self-management) | Can promote patient-centred care by facilitating communication between patients and supporting self-management with provider feedback. | Clinical Effectiveness/ Acceptability | A5. Monitoring condition with feedback |
|  |  | Use of Remote Consultations for a routine asthma review | Prescribing medication using telemedicine technologies, (when a relationship is already established between patient and provider) | Increase in prescribing | The American Medical Association supports prescribing medicine over remote consultations, provided that the patient and health professional already have an existing relationship. | Safety | A6. Practical Support with adherence (medication or behavioural)  A7. Provision of equipment |

| **No.** | **Reference** | **Context – Mechanism - Outcomes Configurations** | | | | Safety/ Clinical Effectiveness/ or Acceptability? | PRISMS Taxonomy Component? |
| --- | --- | --- | --- | --- | --- | --- | --- |
|  |  | Background information e.g., setting and demographics to outline possible **Contextual** factors; | Key workings that contributed to the design and functioning of a pathway to identify **Mechanisms** and resources; | | Information and evidence suggestive of the successes or failures of different aspects of an intervention (**Outcomes**) |  |  |
|  |  |  | Resource | Reaction/Response |  |  |  |
| **6** | Greenhalgh, T., S. Shaw, J. Wherton, S. Vijayaraghavan, J. Morris, S. Bhattacharya, P. Hanson, D. Campbell-Richards, S. Ramoutar and A. Collard (2018). "Real-world implementation of video outpatient consultations at macro, meso, and micro levels: mixed-method study." Journal of medical Internet research 20(4): e150. | Patients with a long-term condition, and a history of defaulting from appointments | Offered their routine review via video conference | Improved patient engagement and attendance rates | Patients showed greater engagement, improved self-management, overall control and a significant reduction in ‘did not attend’ rates. | Clinical Effectiveness | A4. Regular clinical review |
|  |  | Vulnerable or ‘hard to reach’ patients with a long-term condition | Availability of video consultations to request a virtual encounter with a health professional | Improved patient confidence | Allows prompt clinical input, and improved patient confidence in self-management, | Acceptability / Clinical Effectiveness | A8. Provision of easy access to advice or support when needed |

| **No.** | **Reference** | **Context – Mechanism - Outcomes Configurations** | | | | Safety/ Clinical Effectiveness/ or Acceptability? | PRISMS Taxonomy Component? |
| --- | --- | --- | --- | --- | --- | --- | --- |
|  |  | Background information e.g., setting and demographics to outline possible **Contextual** factors; | Key workings that contributed to the design and functioning of a pathway to identify **Mechanisms** and resources; | | Information and evidence suggestive of the successes or failures of different aspects of an intervention (**Outcomes**) |  |  |
|  |  |  | Resource | Reaction/Response |  |  |  |
| **7** | Gruffydd-Jones, K. and S. Ward (2005). "Targeted routine asthma care in general practice using the telephone triage." European respiratory journal 26(Suppl 49): Abstract No. 4264. | People with asthma attending their routine review (within a semi-rural GP Practice) | Availability of telephone consultations to conduct routine review in comparison to face-to-face reviews | Increased patient engagement | Significantly improving access to routine care. Patients are more likely to receive their annual review if conducted via telephone. (35% increase in telephone vs clinic group). | Acceptability | A4. Regular Clinical Review |
|  |  | People with asthma whose routine review is conducted via telephone | Nurse and patient formulise the individualised asthma action plan and advice on what to do if asthma control deteriorated. Written version of the plan is then sent to patient | Increased patient engagement, understanding of their condition and how to recognise deterioration | Increased patient understanding of individual condition, shared decision making between patient and professional and provision of action plan. | Clinical Effectiveness | A3. Provision of/agreement on specific clinical plans and/or rescue medication  A1. Information about condition and /or its management |

| **No.** | **Reference** | **Context – Mechanism - Outcomes Configurations** | | | | Safety/ Clinical Effectiveness/ or Acceptability? | PRISMS Taxonomy Component? |
| --- | --- | --- | --- | --- | --- | --- | --- |
|  |  | Background information e.g., setting and demographics to outline possible **Contextual** factors; | Key workings that contributed to the design and functioning of a pathway to identify **Mechanisms** and resources; | | Information and evidence suggestive of the successes or failures of different aspects of an intervention (**Outcomes**) |  |  |
|  |  |  | Resource | Reaction/Response |  |  |  |
| **8** | Hanlon, P., L. Daines, C. Campbell, B. McKinstry, D. Weller and H. Pinnock (2017). "Telehealth Interventions to Support Self-Management of Long-Term Conditions: A Systematic Metareview of Diabetes, Heart Failure, Asthma, Chronic Obstructive Pulmonary Disease, and Cancer." Journal of medical Internet research 19(5): e172. | Patient with asthma | Provided with a telehealth review (telephone or video consultation) for their routine asthma review (including education support, telemonitoring, provision of action plan) | Positive/Neutral – No differences in patient outcomes between telehealth and face-to-face care for self-management delivery | There are little or no significant differences in the provision of supported self-management components (PRISMS) between remote and face-to-face care for asthma (different for other long-term conditions). Remote care is a safe alternative mode of delivery of self-management support (meta-analysis results). | Clinical Effectiveness | A1. Information about condition and/ or its management  A3. Provision of/ agreement on specific clinical action plans and or/rescue medication  A5. Monitoring of condition with feedback |

| **No.** | **Reference** | **Context – Mechanism - Outcomes Configurations** | | | | Safety/ Clinical Effectiveness/ or Acceptability? | PRISMS Taxonomy Component? |
| --- | --- | --- | --- | --- | --- | --- | --- |
|  |  | Background information e.g., setting and demographics to outline possible **Contextual** factors; | Key workings that contributed to the design and functioning of a pathway to identify **Mechanisms** and resources; | | Information and evidence suggestive of the successes or failures of different aspects of an intervention (**Outcomes**) |  |  |
|  |  |  | Resource | Reaction/Response |  |  |  |
| **9** | Ignatowicz, A., H. Atherton, C. J. Bernstein, C. Bryce, R. Court, J. Sturt and F. Griffiths (2019). "Internet videoconferencing for patient–clinician consultations in long-term conditions: A review of reviews and applications in line with guidelines and recommendations." Digital health 5: 2055207619845831. | A patient with asthma scheduled routine review | Review is conducted via videoconference | Meets patient needs and preferences | Implementation of videoconferences as a means for routine asthma reviews can reduce barriers to treatment and increase convenience for patients. | Acceptability | A4. Regular Clinical Review  A8. Provision of easy access to advice or support when needed |
|  |  | A patient with asthma scheduled routine review | Review is conducted via videoconference and provision of patient education during review | Increased patient individualised understanding and knowledge of condition | Provision of patient education during routine video consultations can increase patient satisfaction and improve health outcomes. | Clinical Effectiveness | A1. Information about condition and/ or its management |
|  |  | A young person with a long-term condition, scheduled for a routine review | Review is conducted via videoconference with the same clinician each time | More personalised care tailored to the young person’s preference and building of relationship with professional | Improved relationship between patient and professional, which lead to more frequent contact with the specific clinician who is known to the patient and likely to know particular young patient’s personal circumstances and what is important to them. | Acceptability / Clinical Effectiveness | A4. Regular Clinical Review  A8. Provision of easy access to advice or support when needed  A9. Training/ Rehearsal to communicate with healthcare professionals |

| **No.** | **Reference** | **Context – Mechanism - Outcomes Configurations** | | | | Safety/ Clinical Effectiveness/ or Acceptability? | PRISMS Taxonomy Component? |
| --- | --- | --- | --- | --- | --- | --- | --- |
|  |  | Background information e.g., setting and demographics to outline possible **Contextual** factors; | Key workings that contributed to the design and functioning of a pathway to identify **Mechanisms** and resources; | | Information and evidence suggestive of the successes or failures of different aspects of an intervention (**Outcomes**) |  |  |
|  |  |  | Resource | Reaction/Response |  |  |  |
| **10** | Kew, K. M. and C. J. Cates (2016). "Remote versus face‐to‐face check‐ups for asthma." Cochrane Database of Systematic Reviews(4). | Patients with asthma, attending a regular routine review | Routine review conducted via remote consultation (telephone or video review, including personalised contact with a health professional) | Increased convenience and engagement of patients in attendance and self-management strategies | Provides an unobtrusive and efficient way of maintaining contact with patients. Remote check-ups may not disrupt a person’s life in the way a regular clinic visit might and may serve to enhance self-management behaviours such as keeping a personalised action plan up to date and adherence to medications. | Acceptability / Clinical Effectiveness | A5. Monitoring of Condition with Regular Feedback  A3. Provision of/ Agreement on specific clinical action plans and/ or rescue medication  A4. Regular clinical review  A6. Practical Support with adherence (medication or behavioural) |

| **No.** | **Reference** | **Context – Mechanism - Outcomes Configurations** | | | | Safety/ Clinical Effectiveness/ or Acceptability? | PRISMS Taxonomy Component? |
| --- | --- | --- | --- | --- | --- | --- | --- |
|  |  | Background information e.g., setting and demographics to outline possible **Contextual** factors; | Key workings that contributed to the design and functioning of a pathway to identify **Mechanisms** and resources; | | Information and evidence suggestive of the successes or failures of different aspects of an intervention (**Outcomes**) |  |  |
|  |  |  | Resource | Reaction/Response |  |  |  |
| **11** | Pinnock, H. (2003). "It's good to talk... but do I really need to see you? The potential of telephone consultations for providing routine asthma care." Primary care respiratory journal 12(3): 79‐80. | A patient with asthma scheduled for a routine asthma review | Provided with a telephone review instead of face-to-face review | Patients are impressed by the convenience of telephone reviews | Telephone reviews help overcome the barrier of access to care. | Acceptability/ Clinical Effectiveness | A4. Regular Clinical Review  A8. Provision of easy access to advice or support when needed |
|  |  | A patient with asthma scheduled for a routine asthma review | Provided with a telephone review instead of face-to-face review and discussed individual medication and self-management with health professional | Patient is provided with knowledge and information of how to manage their individual condition | Patient can be provided with information regarding their asthma and management of their asthma (A1), are able to be signposted to supporting literature/websites for available resources (A2) and can be provided with advice and support around health and lifestyle (A14) e.g., stopping smoking, via telephone consultation. | Safety/ Clinical Effectiveness | A1. Information about condition and/ or its management  A2. Information about available resources  A14. Lifestyle advise and support |

| **No.** | **Reference** | **Context – Mechanism - Outcomes Configurations** | | | | Safety/ Clinical Effectiveness/ or Acceptability? | PRISMS Taxonomy Component? |
| --- | --- | --- | --- | --- | --- | --- | --- |
|  |  | Background information e.g., setting and demographics to outline possible **Contextual** factors; | Key workings that contributed to the design and functioning of a pathway to identify **Mechanisms** and resources; | | Information and evidence suggestive of the successes or failures of different aspects of an intervention (**Outcomes**) |  |  |
|  |  |  | Resource | Reaction/Response |  |  |  |
| **12** | Pinnock, H., L. Adlem, S. Gaskin, J. Harris, C. Snellgrove and A. Sheikh (2007). "Accessibility, clinical effectiveness, and practice costs of providing a telephone option for routine asthma reviews: phase IV controlled implementation study." British journal of general practice 57(542): 714‐722. | A patient with asthma scheduled for a routine asthma review | Provided with a telephone review instead of a face-to-face review | Increased confidence and enablement in individual asthma care | Patients provided with a routine telephone review offer a stable ‘maintenance’ phase of monitoring, during which self-management assumes precedence. In turn, can increase patient’s confidence in managing their own condition. | Clinical Effectiveness | A1.Information about condition and/ or its management  A4. Regular clinical review |
|  |  | Adults with asthma for routine review  (Asthma care was provided by five asthma-trained nurses across the two practice sites. Asthma clinics offered a range of appointment times throughout the week, and these were supplemented  with opportunistic arrangements to suit patient availability) | Telemedicine appointment with nurse | Increase in update of appointment/  Increased confidence and self-management | Cost-effective method. | Clinical Effectiveness/  Acceptability | A4. Regular clinical review |

| **No.** | **Reference** | **Context – Mechanism - Outcomes Configurations** | | | | Safety/ Clinical Effectiveness/ or Acceptability? | PRISMS Taxonomy Component? |
| --- | --- | --- | --- | --- | --- | --- | --- |
|  |  | Background information e.g., setting and demographics to outline possible **Contextual** factors; | Key workings that contributed to the design and functioning of a pathway to identify **Mechanisms** and resources; | | Information and evidence suggestive of the successes or failures of different aspects of an intervention (**Outcomes**) |  |  |
|  |  |  | Resource | Reaction/Response |  |  |  |
| **13** | Raju, J. D., A. Soni, N. Aziz, J. D. Tiemstra and M. Hasnain (2012). "A patient-centered telephone intervention using the asthma action plan." Family medicine 44(5): 348-350. | Patients with clinically diagnosed uncontrolled asthma | Contacted by a health professional via telephone, personalised asthma action plan discussed and developed to adjust medication | Individual asthma control improved (assessed by Asthma Control Score) | Asthma control can not only be assessed via telephone, but also significantly improved when action plan is discussed with healthcare professional during routine review. | Clinical Effectiveness / Acceptability | A1. Information about conditions and / or its management  A3. Provision of/ agreement on specific clinical action plans and/ or rescue medication |
|  |  | Adults with asthma - university-based family medicine residency clinic.  Patients were contacted by phone, and an initial Asthma Control Score (ACS) was assessed. Patients with an ACS <20 (uncontrolled asthma) had their medication adjusted and a new AAP implemented by phone. Uncontrolled patients were reassessed by phone monthly and management was adjusted until control was achieved. | Telemedicine appointments – triaged by Asthma Control Score - with uncontrolled patients getting monthly assessments | Improved asthma control (Asthma Control Score) | 1, targeted telephone care  management programs can be successful in reducing medical costs and hospitalizations  2. constrained by the proportion of patients who are difficult to reach by telephone because they lack functioning  message systems and/or seldom answer their phone when care providers try to call them.  3. From a practical standpoint,  physicians would likely not have the time to call patients for the initial assessment (someone else to do the initial triage) | Clinical Effectiveness | A3. Provision of/agreement on specific clinical action plans and/or rescue medication  A1. Information about conditions and / or its management  A5. Monitoring of condition with feedback |

| **No.** | **Reference** | **Context – Mechanism - Outcomes Configurations** | | | | Safety/ Clinical Effectiveness/ or Acceptability? | PRISMS Taxonomy Component? |
| --- | --- | --- | --- | --- | --- | --- | --- |
|  |  | Background information e.g., setting and demographics to outline possible **Contextual** factors; | Key workings that contributed to the design and functioning of a pathway to identify **Mechanisms** and resources; | | Information and evidence suggestive of the successes or failures of different aspects of an intervention (**Outcomes**) |  |  |
|  |  |  | Resource | Reaction/Response |  |  |  |
| **14** | Van Gaalen, J. L., S. Hashimoto and J. K. Sont (2012). "Tele management in asthma: an innovative and effective approach." Current Opinion in Allergy & Clinical Immunology 12(3): 235-240. | People with asthma | Regular routine asthma review is conducted via telemedicine (telephone/video consultation) | Patient feels empowered and has the tools to be able to manage their condition | Use of telemedicine provides patients with the tools to self-manage and gain control over their condition (due to provision of; self-monitoring (A5), patient is able to detect and respond to symptom worsening (A1) and can easily contact a professional (A8). | Clinical Effectiveness | A1. Information about condition and/ or its management  A5. Monitoring of condition with feedback  A8. Provision of easy access to advice or support when needed |
|  |  | People with asthma | Regular routine asthma review is conducted via telemedicine (telephone/video consultation) and provision/discussions take place about individualised asthma action plan | Patient has an individualised action plan and understands their condition and what to do if symptoms worsen or change | Use of telemedicine enables proactive individual patient care through the provision of a personalised asthma action plan. | Clinical Effectiveness | A3. Provision of/ agreement on specific clinical action plans and/or rescue medication |

| **No.** | **Reference** | **Context – Mechanism - Outcomes Configurations** | | | | Safety/ Clinical Effectiveness/ or Acceptability? | PRISMS Taxonomy Component? |
| --- | --- | --- | --- | --- | --- | --- | --- |
|  |  | Background information e.g., setting and demographics to outline possible **Contextual** factors; | Key workings that contributed to the design and functioning of a pathway to identify **Mechanisms** and resources; | | Information and evidence suggestive of the successes or failures of different aspects of an intervention (**Outcomes**) |  |  |
|  |  |  | Resource | Reaction/Response |  |  |  |
| **15** | Vitacca, M., L. Comini and S. Scalvini (2010). "Is teleassistance for respiratory care valuable? Considering the case for a 'virtual hospital'." Expert Review of Respiratory Medicine 4(6): 695-697. | Patients with asthma | Providing the patient with a routine review using telemedicine (telephone or video consultation) | Patients are supported to manage their asthma more effectively | Results in quick transmission of information and clinical data in real-time, thus leading to greater continuity of care. Can provide active education and support. | Clinical Effectiveness | A1. Information about and/ or its management  A4. Regular clinical review |
|  |  | Patients with asthma | Providing the patient with a routine review using telemedicine (telephone or video consultation) and use of telemonitoring of respiratory measures (e.g., peak expiatory flow) | Patient is able to adjust their medication and detect if symptoms may be worsening | Use of monitoring via telemedicine can result in earlier detections of symptoms exacerbations. As these measures may be missed by a patient who would not visit a practice for a face-to-face review. | Clinical Effectiveness | A4. Regular Clinical Review  A5. Monitoring of condition with feedback  A6. Practical support with adherence (medication or behavioural)  A11. Training/ rehearsal for everyday activities |

| **No.** | **Reference** | **Context – Mechanism - Outcomes Configurations** | | | | Safety/ Clinical Effectiveness/ or Acceptability? | PRISMS Taxonomy Component? |
| --- | --- | --- | --- | --- | --- | --- | --- |
|  |  | Background information e.g., setting and demographics to outline possible **Contextual** factors; | Key workings that contributed to the design and functioning of a pathway to identify **Mechanisms** and resources; | | Information and evidence suggestive of the successes or failures of different aspects of an intervention (**Outcomes**) |  |  |
|  |  |  | Resource | Reaction/Response |  |  |  |
| **16** | **(Added by External Reference Group)** Hamour, O., Smyth, E., & Pinnock, H. (2020). Completing asthma action plans by screen-sharing in video-consultations: practical insights from a feasibility assessment. NPJ primary care respiratory medicine, 30(1), 1-5. | Patients with asthma, scheduled for a routine review | Review conducted via video-consultation and health professional uses the ‘edit document’ and screen sharing features during the video consultation to review asthma action plan | Improved understanding and relationship between patient and professional | Patients felt editing the document with the clinician collaboratively improved communication and avoided misunderstandings. It also enhanced shared decision making between individual and professional. | Clinical Effectiveness/ Acceptability/ Safety | A3. Provision of/ agreement on specific clinical action plans and/ or rescue medication  A9. Training/ rehearsal to communicate with healthcare professionals |
|  |  | Patients with asthma, scheduled for a routine review | Review conducted via video consultation. Consultation is recorded via videoconferencing software | Patients appreciated being able to review their consultation and what had been discussed | Patients can revisit their review and help consolidate the information delivered during to better understand their asthma and how to manage their condition. | Clinical Effectiveness/ Acceptability | A1. Information about condition and/or its management  A9. Training/ rehearsal to communicate with healthcare professionals |
|  |  | Patients with asthma, scheduled for a routine review,= | Review conducted via video consultation and asthma action plan discussed via screen sharing | Patients found the approach to be comparable to live situations | Online screen-sharing is a practical approach to joint completion of asthma action plans. | Clinical Effectiveness/ Acceptability | A3. Provision of/ agreement on specific clinical action plans and/ or rescue medication |

| **No.** | **Reference** | **Context – Mechanism - Outcomes Configurations** | | | | Safety/ Clinical Effectiveness/ or Acceptability? | PRISMS Taxonomy Component? |
| --- | --- | --- | --- | --- | --- | --- | --- |
|  |  | Background information e.g., setting and demographics to outline possible **Contextual** factors; | Key workings that contributed to the design and functioning of a pathway to identify **Mechanisms** and resources; | | Information and evidence suggestive of the successes or failures of different aspects of an intervention (**Outcomes**) |  |  |
|  |  |  | Resource | Reaction/Response |  |  |  |
| **17** | **(Added by External Reference Group)** Pare, G., K. Moqadem, G. Pineau and C. St-Hilaire (2010). Clinical effects of home telemonitoring in the context of diabetes, asthma, heart failure and hypertension: A systematic review. [References], Journal of Medical Internet Research. Vol.12(2), 2010, pp. p190-p204. | Patients with asthma, provided with the equipment and instruction to monitor their condition via telemonitoring | Offered a remote consultation to review condition | Feelings of empowerment & improved feelings of security | Patients are able to actively participate in their own care. | Clinical effectiveness / Acceptability | A5. Monitoring of condition with feedback |
|  |  | Patients with asthma, provided with the equipment and instruction to monitor their condition via telemonitoring | Used an interactive tool via remote consultation to monitor condition and gain feedback from a healthcare professional | Empowerment | Fewer asthma related symptoms, and patient’s asthma was better controlled. | Clinical Effectiveness | A5.Monitoring of condition with feedback  A9. Training/ Rehearsal to communicate with healthcare professionals |

| **No.** | **Reference** | **Context – Mechanism - Outcomes Configurations** | | | | Safety/ Clinical Effectiveness/ or Acceptability? | PRISMS Taxonomy Component? |
| --- | --- | --- | --- | --- | --- | --- | --- |
|  |  | Background information e.g., setting and demographics to outline possible **Contextual** factors; | Key workings that contributed to the design and functioning of a pathway to identify **Mechanisms** and resources; | | Information and evidence suggestive of the successes or failures of different aspects of an intervention (**Outcomes**) |  |  |
|  |  |  | Resource | Reaction/Response |  |  |  |
| **18** | **(Added by External Reference Group)** Thiyagarajan, A., C. Grant, F. Griffiths and H. Atherton (2020). "Exploring patients' and clinicians' experiences of video consultations in primary care: a systematic scoping review." BJGP open 4(1). | Patients with asthma, (most specifically for older patients) | Routine consultation conducted via video consultation | Patient benefits: increased patient convenience, reduced travel costs | Improved access to support. | Acceptability | A8. Provision of easy access to advice or support when needed |
